# Effects of a powder made from three medicinal plants on growth performance, intestinal health, antioxidant activity, and anti-inflammatory ability in Xianghuang chickens

**DOI:** 10.3389/fvets.2025.1538623

**Published:** 2025-03-26

**Authors:** Can Yang, XiaoWu Tang, RunTao Wu, YunMiao Jiang, Qi Quan, YuTian Xiao, JiaXuan Kuang, JiaYi Chen, QingHai Tang, Zhi Jiang

**Affiliations:** ^1^College of Life Sciences, Hunan Provincial Key Laboratory of Biological Resources Protection and Utilization in NanYue Mountain Area, Hengyang Normal University, Hengyang, China; ^2^College of Bioengineering, Hunan Vocational Technical College of Environment and Biology, Hengyang, China; ^3^Hunan Provincial Key Laboratory of the Traditional Chinese Medicine Agricultural Biogenomics, Changsha Medical University, Changsha, China; ^4^Yimin Ecological Agriculture Development Co., LTD, Hengyang, China

**Keywords:** Chinese medicinal plant, antioxidant function, intestinal health, chickens, anti-inflammation

## Abstract

This study investigated the effect of a traditional Chinese medicine (TCM) plant powder made from an equal proportion of *Sarcococca ruscifolia* Stapf, *Hedera nepalensis var. sinensis* (Tobl.) Rehd, and *Clematis chinensis* Osbeck on growth performance and intestinal health in Xianghuang chickens, focusing on intestinal histomorphology, antioxidant activity, and anti-inflammation function. A total of 100 10-day-old male Xianghuang chickens were randomly assigned to two groups, with five replicate cages per group containing 10 birds each. The birds in the control group received a corn-soybean–based diet, while the birds in the TCM group received the control diet supplemented with 2% of the TCM powder. The chickens were slaughtered for sample collection on D28. The results showed that the average daily feed intake (ADFI), average daily gain (ADG), and feed-to-gain (F:G) ratio were not affected by the TCM supplementation (*p* > 0.05). In the jejunum and ileum, the ratio of the villus height to the crypt depth was higher in the TCM group compared to the control group (*p* < 0.05). Supplementing the chickens with 2% TCM powder increased the total superoxide dismutase (T-SOD) and catalase (CAT) activities in the jejunal mucosa compared to the control group (*p* < 0.05). The gene expression of tumor necrosis factor alpha (TNF-α) was downregulated in the jejunal mucosa and spleen in the TCM group compared to the control group (*p* < 0.05). In conclusion, TCM powder can be safely utilized to promote the development of the intestinal tract by enhancing antioxidant and anti-inflammatory functions without affecting the growth performance. Our findings suggest that TCM powder is an effective and low-toxicity natural additive for intestinal improvement in poultry.

## Introduction

Increasing consumer demand for antibiotic-free poultry products has driven the search for effective and safe additives in poultry production. Traditional Chinese medicinal plants are promising antibiotic substitutes and have been utilized in livestock for centuries. *Sarcococca ruscifolia* Stapf belongs to the Buxaceae family and is rich in pachysandra alkaloids, which have been shown to inhibit HepG2 cell migration and induce mitochondria-mediated intrinsic apoptosis (1). These pachysandra alkaloids contain cytotoxic pregnane derivatives that are effective against melanoma and lung cancer cells (2). Plants from *Sarcococca* Lindl. have anti-inflammatory (3) effects. The crude extract of *Sarcococca saligna* Muel showed dose-dependent antidiarrheal activities against castor oil-induced diarrhea in mice at doses of 500 and 1,000 mg/kg (4).

*Hedera helix* (ivy) belongs to the Araliaceae family, and its leaves contain saponins such as monodesmoside α-hederin and hederacoside (5), which have demonstrated antibacterial and anti-inflammatory effects (6). *Hedera* species exhibited validated anti-inflammatory effects in ovalbumin-sensitized guinea pigs through the modulation of immune cell populations, such as a decrease in total white blood cell and eosinophil counts but an increase in neutrophils, lymphocytes, and monocytes (7).

*Clematis chinensis* Osbeck (*C. chinensis*) belongs to the Ranunculaceae family and has been extensively utilized for its root-derived preparations, which exhibit anti-inflammatory, antitumor, and analgesic properties (8). Novel phenolic glycosides and alkaloids have been isolated from the roots and rhizomes of C. *chinensis*; they displayed inhibitory effects on nitric oxide (NO) production and anti-inflammatory activity in lipopolysaccharide (LPS)-induced RAW 264.7 cells (9). Total saponins extracted from *C. chinensis* exhibited significant anti-inflammatory effects in both cellular and animal models. Oral administration of 100 mg/kg of saponin extract effectively attenuated inflammatory responses in LPS-challenged rabbits and primary human chondrocytes (10).

Previous studies have focused on *in vitro* or cellular experiments. To date, there have been few documented cases of the direct application of botanical additives in animal production practices. Our study revealed the presence of bioactive components, including polysaccharides, total flavonoids, polyphenols, and total protein, in an aqueous extract of a TCM plants mixture consisting of *Sarcococca ruscifolia* Stapf, *Hedera nepalensis var. sinensis* (Tobl.) Rehd, and *C. chinensis*. Notably, previous research has shown that flavonoid- and polysaccharide-enriched extracts from *Astragalus* spp. effectively mitigate *E. coli* infection in poultry (11). Extraction is a time-consuming process in itself, and the extractive organic solvents such as petroleum and ethanolic are also toxic. Historical documentation in the Compendium of Materia Medica (12) classifies plants such as *Sarcococca ruscifolia* Stapf, *Hedera nepalensis var. sinensis* (Tobl.) Rehd, and *C. chinensis* as non-toxic, despite their characteristic bitter taste. Safety assessments, corroborated by modern pharmacological evidence, show the therapeutic efficacy of *Hedera helix* (Ivy leaf) in managing respiratory symptoms (13). Xianghuang broilers have gained attention in poultry research due to their high-quality meat and adaptability. They are local, yellow-feathered, slow-growing breed found in southern China. Poultry, including Xianghuang broilers, generally have a low tolerance for bitter compounds and exhibit moderate tolerance to dietary fiber. It remains unclear whether chickens can tolerate the fiber and bitterness of these TCM plants without affecting their production performance. Based on these findings, we hypothesized that supplementation with these TCM plants may safely enhance gastrointestinal health in Xianghuang broilers through antioxidant-mediated epithelial barrier protection and immunomodulation of gut-associated lymphoid tissue responses, without affecting growth performance. This study may help identify highly effective and low-toxicity natural anti-inflammatory plant products from Chinese medicine.

## Materials and methods

### Preparation of traditional Chinese medicine (TCM)

Fresh plants were harvested from the mountainous area of ShaoYang, Hunan, China, based on advice from local older individuals. The plants, including the root, stem, and leaves, were sun-dried for five to seven days under strong sunlight on the ground. The plants were then cut into 5–10 cm-long segments using a chainsaw. The plant segments were sun-dried again, crushed using a traditional Chinese medicine grinder, and sieved through a 60-mesh (ø250 μm) screen. The powders of *Sarcococca ruscifolia* Stapf, *Hedera nepalensis var. sinensis (Tobl.) Rehd*, and *Clematis chinensis Osbeck* were mixed in a ratio of 1:1:1 to form the TCM mixture. The nutrient contents of the TCM plant powder, based on air-dried matter, were as follows: dry matter 90.98 ± 0.60%, total protein 13.81 ± 2.41%, crude fat 0.29 ± 0.05%, crude fiber 32.85 ± 0.50%, Ca 0.24 ± 0.04%, total phosphorus 0.14 ± 0.01%, and crude ash 3.95 ± 0.99%. Active ingredients, including polysaccharides (concentration 1.26 mg/mL, extraction rate 0.99%), total flavonoids (concentration 0.07 mg/mL extraction rate 0.57%), polyphenols (concentration 0.09 mg/mL, extraction rate 0.35%), and total protein (concentration 0.09 mg/mL, extraction rate 0.07%), were identified in the plant water extracts.

### Animals and treatment

The research was approved by the Animal Care and Use Committee at Hengyang Normal University under project number HNUACUC-B202111001.

A total of 100 10-day-old male native Xianghuang broilers, with an average initial body weight of 99.33 ± 4.41 g, were randomly assigned to two replicated experimental groups: control (C) and experimental (E), with five replicates per group and 10 birds per replicate. A corn-soybean-based diet was formulated to exceed the nutritional requirements of the yellow-feathered broilers (14) (Table 1) and was fed to the birds in the control group. The birds in the experimental group were supplemented with 2% of the TCM plant powder in the control diet. All birds were housed in pens (0.10 m^2^/bird) with padded rice hulls located at Yimin Farm and had ad libitum access to water and feed. The temperature of the room was maintained at around 29.0°C for the first week (when the broilers were 11 days old) and then gradually reduced by 3°C every week to 24°C until the end of the study using an air conditioner. The light was provided for 12 h at 20 lux using LED lights throughout the experimental period.

**Table 1:** Composition and nutrient levels of the basal diet (as-fed basis) %.

Items	Content
Ingredients	
Corn	53.79
Soybean meal	39.08
Soybean oil	2.80
Limestone	1.02
CaHPO_4_	1.79
Premix^1^	1.00
NaCl	0.34
Met	0.18
Total	100.00
Nutrient levels^2^, %	
Metabolic energy/(MJ/kg)	12.14
Crude fiber	2.30
Crude fat	5.40
Crude protein	21.00
Ca	0.90
Total phosphorus (P)	0.68
Digestible P	0.47
NaCl	0.37
Lys	1.08
Met+Cys	0.85
Thr	0.89
Trp	0.30

1 The premix provided the following per kilogram of the diet: retinyl acetate 12,500 IU, cholecalciferol 3,500 IU, *DL*-alpha tocopheryl acetate 25 IU, riboflavin 8.5 mg, VB_12_ 30 μg, *D*-calcium pantothenate 15 mg, niacin 30 mg, folic acid 1 mg, biotin 0.1 mg, choline chloride 509 mg, Cu 8 mg, Fe 80 mg, Mn 110 mg, Zn 110 mg, Se 0.15 mg, and I 0.35 mg.

2 Nutrient levels were all calculated values, and amino acids were standardized to ileal digestible amino acids.

### Growth performance determination

All broilers were individually weighed after 12 h of feed deprivation. The body weight (BW) and feed leftovers were recorded per replicate weekly through the experiment. The average daily feed intake (ADFI), average daily gain (ADG), and feed-to-gain (F:G) ratio (the ratio of ADFI to ADG) were calculated.

### Sample collection

On day 28, 10 broilers were randomly selected from each of the control and experimental groups for sample collection. Blood was individually collected via the wing vein using a 22 g needle, a venous blood collection needle, and a red vacuum blood collection tube. After standing at room temperature for 3 h, 5 mL of blood was centrifuged at 3,000 rpm at 4°C for 15 min. The supernatants (serum) were collected and stored at −80°C until further analysis. After that, the broilers were euthanized by carbon dioxide followed by cervical dislocation. Organ samples, including the liver, spleen, intestine, kidney, bursa, and heart, were weighed, and the organ index was calculated as follows, Organ index = Organ weight*100%/live body weight. Approximately 5 cm segments of the proximal duodenum, mid jejunum, and distal ileum were washed with 0.9% saline to remove the intestinal contents and then carefully fixed in 4% paraformaldehyde for 48 h for histological examination. The remaining segments of the small intestine were washed with 0.9% sterile saline and scraped with a glass slide to collect the mucosa. The mucosa and spleen samples were frozen in liquid nitrogen and then stored at −80°C for further analysis.

### Histological investigation

The intestinal segments fixed in 4% paraformaldehyde were dehydrated with increasing concentrations of ethanol, vitrified with dimethylbenzene, embedded in paraffin, and then cut into 5-μm-thick slides. These were stained with hematoxylin and eosin according to the method mentioned in the report (15). An image of the villus was captured using a microscope (Leica RM2135, Wetzlar, Germany). The width of the baseline, villus height, villus width, and crypt depth of the duodenum, jejunum, and ileum were measured from 50 villi per broiler using Image-Pro Plus (Media Cybernetics Inc., Silver Spring, MD, USA).

### Antioxidant indices analysis

Antioxidant parameters, including the activities of T-SOD, CAT, total antioxidant activity (T-AOC), and malonaldehyde (MDA) concentration in the serum and the supernatants of the mucosal samples, were determined according to the method mentioned in the report (15, 16). Approximately 0.1 g of the mucosa was homogenized with 0.9 mL of 0.9% sterile saline using a tissue grinder (SCIENTZ-12, Xinzhi Biotech logy, Ningbo, China) and then centrifuged at 2,500 r/min for 15 min to collect the supernatant. The activity of T-AOC (mmol/L) was analyzed using the OD 593 nm value of the supernatant compared with a standard curve of FeSO_4_.7H_2_O. The unit of CAT was defined as the amount of hydrolyzed H_2_O_2_ in 1 min per mg of protein in the sample. One unit of T-SOD was defined as the amount that caused 50% inhibition of the nitroblue tetrazolium light reaction rate. The supernatant was extracted with 10% trichloroacetic acid and then used to test MDA concentration.

### RT-PCR analysis

The mRNA expression levels of inflammation, intestinal barrier function, and apoptosis-related genes were examined using quantitative real-time PCR. Total RNA from the mucosa and spleen samples was isolated using TRIzol Reagent (Invitrogen, Carlsbad, CA, USA). The integrity of the RNA was determined by 2% agarose gel electrophoresis. The concentration and purity of the RNA were measured using the NanoDrop ND-2000 spectrophotometer system (Thermo Fisher Scientific). One μg of the total RNA was reverse transcribed into DNA using an RT reagent kit (Aikerui, Changsha, China) according to the manufacturer’s instructions. The RT-PCR was performed on a QuantStudio3 device (Thermo Fisher Scientific, Waltham, MA, USA) using a SYBR Green quantitative PCR mix (Aikerui, Changsha, China) following the manufacturer’s protocols. The primer sequences for the target and reference (β-actin) genes are listed in Table 2. The mRNA expression levels of the target genes were calculated using the 2^−ΔΔCT^ method (17).

**Table 2:** Sequences of the primers used for the quantitative real-time PCR.

Gene	Size (pb)	Accession N°	Sequence (5^′^–3^′^)
β-Actin (β-actin)	108	NM_205518.1	F: CATTGTCCACCGCAAATGCT R: AGCCATGCCAATCTCGTCTT
TNF-α (tumor necrosis factor-α)	113	MF000729.1	F: GGGACGGCCTTTACTTCGTA R: GTCTTTGGGGTACTCCTCGG
IL-1β (interleukin-1β)	204	NM_204524.1	F: TGCCTGCAGAAGAAGCCTCG R: GACGGGCTCAAAAACCTCCT
IL8L2 (interleukin-8)	174	NM_205498.1	F: CCTAACCATGAACGGCAAGC R: CTTGGCGTCAGCTTCACATC
TLR4 (toll-like receptor 4)	171	NM_001030693.1	F: TGACCTACCCATCGGACACT R: CTCAGGGCATCAAGGTCTCC
BCL2 (B-cell lymphoma-2)	128	NM_205339.2	F: GCTGCTTTACTCTTGGGGGT R: CTTCAGCACTATCTCGCGGT
CLDN1 (claudin-1)	294	NM_001013611.2	F: TATGGCAACAGAGTGGCTCG R: TCAGGACAGCGGCATTGTAG

### Statistical analysis

The data were analyzed using the one-way ANOVA procedure of SAS 8.0. The individual broiler was the experimental unit for all data, except for growth performance, where the replicate was the experimental unit. Differences between the treatments were evaluated using a *t*-test. The data were presented as means ± standard deviation. In this study, a *p*-value <0.05 was considered significant, and 0.05 ≤ *p* ≤ 0.10 was considered a tendency.

## Results

### Growth performance

The effect of the TCM supplementation on the growth performance of the Xianghuang broilers is shown in Table 3. No significant differences in the ADFI, ADG, and F:G ratio between the control and experimental groups were observed (*p* > 0.05).

**Table 3:** Effect of the traditional Chinese medicine (TCM) supplementation on the growth performance of the Xianghuang chickens.

Item	Control	TCM	*p*-value
Initial BW, g	96.81 ± 3.31	101.86 ± 4.17	0.107
Final BW, g	399.41 ± 19.59	419.24 ± 10.51	0.125
Week 1			
ADFI, g/bird	19.79 ± 1.29	20.63 ± 1.24	0.384
ADG, g/ bird	9.49 ± 2.18	7.99 ± 3.03	0.451
F:G	2.16 ± 0.49	2.82 ± 0.84	0.224
Week 2			
ADFI, g/bird	22.49 ± 2.62	21.55 ± 3.84	0.701
ADG, g/ bird	7.95 ± 3.97	10.05 ± 5.14	0.542
F:G	3.76 ± 2.62	2.82 ± 2.08	0.595
Week 3			
ADFI, g/bird	25.63 ± 1.64	27.03 ± 1.44	0.249
ADG, g/ bird	10.84 ± 2.46	12.16 ± 2.53	0.484
F:G	2.45 ± 0.50	2.30 ± 0.51	0.697
Week 4			
ADFI, g/bird	49.48 ± 2.20	50.80 ± 0.42	0.283
ADG, g/ bird	15.07 ± 0.81	15.77 ± 0.97	0.309
F:G	3.29 ± 0.22	3.23 ± 0.22	0.724
Total 4 weeks			
ADFI, g/bird	29.63 ± 0.82	30.47 ± 0.85	0.204
ADG, g/ bird	10.81 ± 0.61	11.34 ± 0.26	0.164
F:G	2.75 ± 0.13	2.69 ± 0.11	0.516

BW, body weight; ADFI, average daily feed intake; ADG, average daily gain; F:G, feed:gain.

### Organ weight and organ indices

The TCM plant powder supplementation had no significant effect on the weight and indices of the liver, intestine, kidney, bursa, spleen, and heart (*p* > 0.05) (Table 4).

**Table 4:** Effect of the traditional Chinese medicine (TCM) supplementation on the relative organ indices of the Xianghuang chickens.

Item	Control	TCM	*p*-value
Body weight, g	431.25 ± 44.22	468.75 ± 63.56	0.192
Length of intestine, cm	78.13 ± 11.39	77.63 ± 7.21	0.918
Relative organ indices, % Body weight			
Liver	3.19 ± 1.01	2.96 ± 0.56	0.581
Spleen	0.26 ± 0.05	0.22 ± 0.04	0.089
Intestine	5.86 ± 1.08	5.31 ± 0.31	0.187
Kidney	0.20 ± 0.05	0.20 ± 0.11	0.929
Heart	0.91 ± 0.20	0.85 ± 0.09	0.478
Bursa	0.52 ± 0.14	0.53 ± 0.09	0.854

### Intestinal morphology

**Figure 1:**
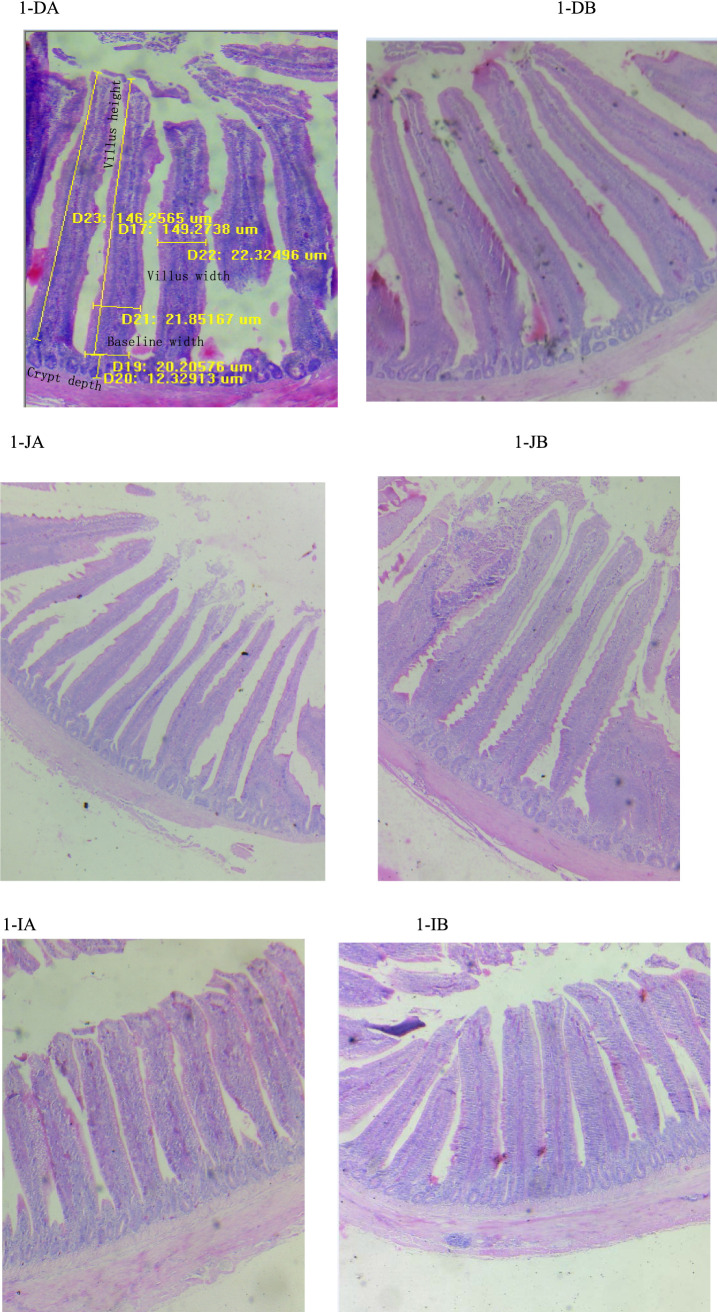
Influence of the traditional Chinese medicine (TCM) supplementation on the intestinal histology of the Xianghuang chickens. A magnification of 10*4 was used. Duodenal villi in the control **(DA)** and TCM **(DB)** groups, jejunal villi in the control **(JA)** and TCM **(JB)** groups, and ileal villi in the control **(IA)** and TCM **(IB)** groups.

The effects of the TCM supplementation on the small intestinal morphology of the broilers are presented in Table 5 and Figure 1. The duodenal villus height exhibited an increasing tendency in the TCM group compared to the control group (*p* = 0.092). However, the baseline width, villus width, and crypt depth of the duodenum remained unaffected by the treatment (*p* > 0.05) (Figures 1DA,DB). The supplementation with TCM notably augmented the baseline and villus widths in the jejunal segment compared to the control group (*p* < 0.05). Within the jejunal segment, there was an increasing trend toward the villus height (*p* = 0.068) and the ratio of the villus height to the crypt depth (*p* = 0.061) in the TCM group compared to the control group (Figures 1JA,JB). The birds in the TCM group had higher villus height and a higher ratio of the villus height to the crypt depth in the ileum compared to their control counterparts (*p* < 0.05). Nonetheless, the crypt depth in both the jejunum and ileum, along with the baseline and villus widths in the ileum, did not exhibit significant alterations due to the TCM treatment (*p* > 0.05) (Figures 1IA,IB).

**Table 5:** Effect of the traditional Chinese medicine (TCM) on the intestinal morphology of the Xianghuang chickens.

Item	Control	TCM	*p*-value
Duodenum, μm			
Baseline width	108.59 ± 21.90	156.12 ± 11.92	0.288
Villus height (VH)	431.43 ± 94.12	578.60 ± 177.62	0.092
Villus width	71.03 ± 20.68	114.14 ± 66.64	0.155
Crypt depth (CD)	58.34 ± 28.19	78.20 ± 29.41	0.228
VH/CD	8.53 ± 3.53	7.59 ± 0.93	0.477
Jejunum, μm			
Baseline width	174.96 ± 36.88	220.74 ± 36.00	0.025
Villus height (VH)	960.30 ± 139.72	1,089.47 ± 121.17	0.068
Villus width	111.04 ± 16.12	137.62 ± 20.16	0.011
Crypt depth (CD)	147.92 ± 33.88	128.84 ± 16.63	0.175
VH/CD	6.88 ± 2.13	8.53 ± 1.02	0.061
Ileum, μm			
Baseline width	215.22 ± 37.67	207.10 ± 40.97	0.686
Villus height (VH)	512.87 ± 150.22	665.79 ± 66.35	0.020
Villus width	183.33 ± 40.44	173.49 ± 30.05	0.589
Crypt depth (CD)	129.90 ± 50.59	110.78 ± 11.01	0.314
VH/CD	4.41 ± 1.69	6.04 ± 0.58	0.022

### Antioxidant capacity

As shown in Table 6, the activities of T-AOC, SOD, and CAT and the concentration of MDA in the ileal mucosa and serum were not affected by the treatment (*p* > 0.05). T-AOC activity and MDA concentration were not affected by the treatment in the jejunal mucosa (*p* > 0.05); however, the jejunal mucosa from the TCM group had higher activities of SOD and CAT compared to the control group (*p* < 0.05).

**Table 6:** Effect of the traditional Chinese medicine (TCM) supplementation on the antioxidant indices of the Xianghuang chickens.

Items	Control	TCM	*p*-value
Serum			
MDA, nmol/mg prot.	3.94 ± 0.54	3.39 ± 0.93	0.193
T-AOC, mmol/mg prot.	2.62 ± 2.34	2.53 ± 0.61	0.928
SOD, U/mg prot.	37.21 ± 22.56	40.77 ± 25.01	0.693
CAT, U/mg prot.	2.82 ± 2.02	3.24 ± 1.68	0.663
Jejunal mucosa			
MDA, nmol/mg prot.	1.92 ± 1.81	0.91 ± 0.72	0.197
T-AOC, mmol/mg prot.	60.24 ± 29.26	57.66 ± 40.51	0.886
SOD, U/mg prot.	256.73 ± 76.55	438.42 ± 216.75	0.044
CAT, U/mg prot.	2.87 ± 1.50	6.26 ± 3.01	0.033
Ileal mucosa			
MDA, nmol/mg prot.	0.67 ± 0.35	0.74 ± 0.61	0.812
T-AOC, mmol/mg prot.	35.07 ± 24.69	28.30 ± 16.18	0.536
SOD, U/mg prot.	132.44 ± 71.40	147.22 ± 103.46	0.756
CAT, U/mg prot.	2.49 ± 1.17	1.73 ± 1.23	0.241

MDA, malonaldehyde; T-AOC, total antioxidant capacity; SOD, superoxide dismutase; TP, total protein; CAT, catalase.

### Expression of the genes

**Figure 2:**
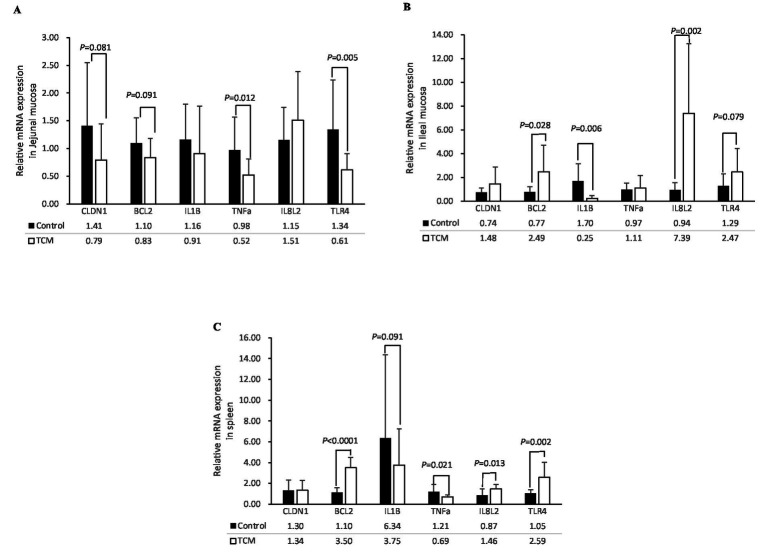
Influence of the traditional Chinese medicine (TCM) supplementation on the relative mRNA expression of the barrier function and inflammation-related genes in the jejunal mucosa **(A)**, ileal mucosa **(B)**, and spleen **(C)** of the Xianghuang chickens. The data are expressed as means ± standard error. The significant differences between the groups are indicated by *p*-values. TNF-a, tumor necrosis factor-a; IL-1β, interleukin-1β; IL8L2, interleukin-8; TLR4, toll-like receptor 4; BCL2, B-cell lymphoma-2; CLDN1, claudin-1.

The mRNA expression levels of the genes associated with apoptosis, inflammation, and tight junctions in the jejunal mucosa, ileal mucosa, and spleen are shown in Figure 2. In the jejunal mucosa, the expression of the genes tumor necrosis factor alpha (TNF-α) and toll-like receptor 4 (TLR4) was significantly downregulated in the birds supplemented with the TCM plant powder (*p* < 0.05). In addition, there was a tendency for lower expression of the claudin-1 (CLDN1) and B-cell lymphoma-2 (BCL2) genes (*p* = 0.081 and 0.091, respectively) in the TCM group compared to the control group. The expression of the IL-1β gene was not affected by the treatment (*p* > 0.05) (Figure 2A).

In the ileal mucosa, the expression of the BCL2 and IL-8 L2 genes was significantly upregulated (*p* < 0.05) in the TCM group compared to the control group. The Ileal TLR4 gene tended to be higher (*p* = 0.079) in the TCM group compared to the control group (*p* < 0.05). Interestingly, the mRNA expression of the IL-1β gene was lower in the TCM group compared to the control group (*p* < 0.05). No significant differences were observed in the expression of the CLDN1 and TNF-α genes in the ileal mucosa between the two groups (*p* > 0.05) (Figure 2B).

In the spleen, upregulation of BCL2 (*p* < 0.01), IL8L2 (*p* < 0.05), and TLR4 (*p* < 0.01) was observed in the TCM group compared to the control group. The mRNA expression of TNF-α (*p* < 0.05) was downregulated, and the expression of IL-1β (*p* = 0.091) tended to be lower in the TCM group compared to the control group. No effect was observed for the CLDN1 expression in the spleen (*p* > 0.05) (Figure 2C).

## Discussion

Growth performance was not affected by the TCM supplementation. A previous review pointed out that H. *helix* is effective in reducing cough and respiratory problems while being well-tolerated in patients (5). Several studies on Clematidis Radix et Rhizome (CRR), such as *C. chinensis*, have verified its effect in treating diseases, such as relieving rheumatism pain and treating hepatic carcinoma and gastrointestinal issues. However, long-term administration of excessive amounts of CRR was found to cause severe diarrhea and death in mice (18). The present study confirmed that the 2% TCM plant powder was very well tolerated as the birds in the TCM group had the same growth performance and organ index as those in the control group.

Intestinal health was significantly enhanced by the TCM supplementation, as evidenced by a notable increase in the villus height-to-crypt depth ratio in both the jejunum and ileum of the birds in the TCM group. The TCM supplementation demonstrated significant anti-inflammatory effects, which contributed to this improvement. Toll-like receptor 4 (TLR4), a critical mediator of inflammatory responses, is known to promote inflammation and exacerbate tissue damage (19). In chickens with diarrhea, the expression of TLR4 mRNA was elevated in intestinal tissues (11). However, in the present study, the TCM supplementation significantly reduced the TLR4 mRNA expression in the jejunum, highlighting its potential to mitigate inflammation. Similarly, *Hederacoside C* supplementation downregulated genes associated with pro-inflammatory cytokines, such as TNF-α, IL-1β, and IL-6, while upregulating the anti-inflammatory cytokine IL-10 in *S. aureus-*induced RAW 264.7 cells and mice (20). In line with these findings, the present study confirmed that the TCM supplementation inhibited the gene expression of IL-1β while promoting the expression of IL8L2 in the ileal mucosa. Furthermore, TCM may confer significant benefits for villous growth through enhanced activities of SOD and CAT. As critical antioxidant enzymes, SOD facilitates the conversion of superoxide radicals (O2^−^) to hydrogen peroxide (H_2_O_2_) (21), while CAT serves as a primary cellular defense mechanism by decomposing H2O2 into water and oxygen (22). The TCM used in this study contained bioactive constituents such as total flavonoids and polyphenols, which exhibit antioxidant properties. Black cumin (*Nigella sativa* L.), a plant from the Ranunculaceae family, has demonstrated potent antioxidant effects by upregulating key enzymes, including SOD, CAT, and glutathione (GSH) (23). It has also shown protective efficacy against aflatoxin-induced toxicity in ducks (24) and broilers (25). The present study demonstrated that the TCM supplementation significantly upregulated the expression of the anti-apoptosis gene Bcl2 in both the spleen and ileal mucosa. As Bcl2 protein is known to suppress apoptosis (26), this enhancement likely promotes villous cell survival as well. Similarly, steroidal alkaloids isolated from the root of *Sarcococca hookeriana* (Buxaceae) have demonstrated potent inhibitory effects on cancer cell proliferation (27). Chemically synthesized pachysandra alkaloids, following the molecular formula of *Sarcococca ruscifolia* alkaloids, have shown superior anticancer activity against HepG2 cells by regulating the JAK2/STAT3 pathway (1). Berberine, extracted from the roots of Ranunculaceae family plants such as *Hydrastus carnadensis* and *Coptis rhizomes* (28), has also exhibited notable anticancer properties, as evidenced by preclinical studies (29). The anti-apoptotic activity of this TCM may be attributed to the inclusion of *Clematis chinensis* Osbeck and *Sarcococca ruscifolia* Stapf, which are rich in berberine and steroidal alkaloids, respectively. Notably, gastrointestinal tract syndrome is characterized by primary lesions in gut microvascular endothelial apoptosis, and preserving endothelial integrity through apoptosis inhibition has been identified as a critical therapeutic target (30). The anti-inflammatory, antioxidant, and anti-apoptosis properties of the TCM appear to be key mechanisms through which it exerts its effects in stimulating intestinal villus proliferation and mucosal integrity. Furthermore, the observed optimization of intestinal morphology may confer adaptive advantages to poultry chicks under extreme environmental stressors. Its broader implications for juvenile livestock development—particularly in enhancing nutrient assimilation and metabolic resilience—represent a critical area for translational research.

## Conclusion

In conclusion, the combination of *Sarcococca ruscifolia* Stapf, *Hedera nepalensis var. sinensis* (Tobl.) Rehd, and *Clematis chinensis* Osbeck is safe for broiler production. This TCM plant powder has also been demonstrated to effectively regulate intestinal histopathological changes through its anti-apoptosis, anti-inflammatory, and antioxidant properties in chickens.

## Data Availability

The raw data supporting the conclusions of this article will be made available by the corresponding author.
